# Rapid Development of an Efficacious Swine Vaccine for Novel H1N1

**DOI:** 10.1371/currents.RRN1123

**Published:** 2009-10-29

**Authors:** Ryan Vander Veen, Kurt Kamrud, Mark Mogler, Alan T. Loynachan, Jerry McVicker, Peter Berglund, Gary Owens, Sarah Timberlake, Whitney Lewis, Jonathan Smith, DL Hank Harris

**Affiliations:** ^*^Harrisvaccines, Inc.; ^†^AlphaVax, Inc; ^‡^Harrisvaccines Inc., Ames, IA; ^§^University of Kentucky, Livestock Disease Diagnostic Center; ^#^Alphavax Human Vaccines; ^**^Alphavax; ^††^AlphaVax Human Vaccines; ^§§^AlphaVax and ^¶¶^Iowa State University

## Abstract

Recombinant hemagglutinin (HA) from a novel H1N1 influenza strain was produced using an alphavirus replicon expression system. The recombinant HA vaccine was produced more rapidly than traditional vaccines, and was evaluated as a swine vaccine candidate at different doses in a challenge model utilizing the homologous influenza A/California/04/2009 (H1N1) strain. Vaccinated animals showed significantly higher specific antibody response, reduced lung lesions and viral shedding, and higher average daily gain when compared to non-vaccinated control animals. These data demonstrate that the swine vaccine candidate was efficacious at all of the evaluated doses.

## Introduction

    The recent outbreak of pandemic or novel H1N1 in the global human population highlights the zoonotic potential of influenza viruses. The current novel H1N1 virus has been shown to be of swine origin[1].   Even before this current pandemic, numerous cases of zoonotic transmission of swine influenza viruses to humans have been identified.   A review of the literature in 2006 identified 37 civilian cases and 13 military cases of human influenza associated with swine influenza strains, spanning from 1958-2005[2].  Fourteen percent of these cases were documented as fatal.  A more recent study reviewed reported cases of triple-reassortant swine influenza subtype H1 in humans from 2005-February 2009[3].  They found 11 sporadic cases, and all 11 patients recovered after showing clinical influenza symptoms.  Nine of these 11 patients had known exposure to pigs, most of which were ill, either at agricultural fairs or at hog farms. These results mirror studies showing increased antibody titers to swine influenza viruses among hog farm workers and family members[4] [5].  This demonstrates the role that human and swine interaction can play in the creation of novel influenza viruses, and thus the need for efficacious swine influenza vaccines.  Currently, novel H1N1 influenza has been detected in swine in several different countries, including Canada, Argentina, Australia, Singapore, United Kingdom, Ireland, Norway, and Japan.[6] [7].  Recently, the novel H1N1 appeared in the United States in a pig exhibited at a fair in Minnesota[8].  Thus, the need for an efficacious novel H1N1 swine vaccine is evident.  Novel H1N1 vaccination can reduce clinical disease in pigs, and may reduce transmission among the swine population and decrease the zoonotic potential.

    Many vaccines have been evaluated using alphavirus replicon technology (reviewed in [9]).  In this study, the alphavirus replicon is derived from the TC-83 strain of the alphavirus Venezuelan Equine Encephalitis Virus (VEEV).  In a previous study, a VEEV replicon vaccine expressing the HA gene from a human H5N1 isolate protected chickens from lethal challenge[10].  Recently, our group became the first to evaluate VEEV replicon particle vaccines in swine[11].  However, no studies have been published using replicon-expressed recombinant proteins as vaccine candidates for swine.  The objective of this study was to evaluate replicon-expressed recombinant novel H1N1 HA protein as a swine vaccine in a vaccination-challenge model. 

## Materials and Methods

### Novel H1N1 HA Replicon Subunit Vaccine Production

The  Influenza A/California/04/2009 hemagglutinin (HA) nucleotide sequence was retrieved from the Global Initiative on Sharing Avian Influenza Data (GISAID) database. The gene was synthesized by a commercial company (DNA2.0, Menlo Park, CA, USA) with unique AscI and PacI restriction sites engineered at the 5’ and 3’ ends, respectively.  The HA gene was cloned into the AscI/PacI sites of the pVEK (TC-83) replicon vector[12] and an optimized construct was selected as previously described[13].  The HA gene was then sequenced to ensure the proper sequence was maintained through the cloning process.  RNA transcripts were produced *in vitro* as previously described[13].  Replicon RNA was mixed with Vero cells in electroporation cuvettes and pulsed.  Cells were incubated overnight and then lysed using RIPA buffer (Pierce, Rockford, IL, USA).  Resulting lysate was tested for protein expression by Western blot and HA protein concentration was determined by a novel H1N1 HA-specific ELISA.   Lysate was diluted to the specified HA concentration and vaccine was adjuvanted with Emulsigen-D (MVP Technologies, Omaha, NE, USA).

### Western Blot Analysis

Vero cell lysate containing recombinant HA protein was separated by running on a 12% SDS-PAGE gel (Invitrogen, Carlsbad, CA, USA) and was then transferred to a PVDF membrane (Invitrogen, Carlsbad, CA).  The  ladder used was the SeeBlue Plus2 Pre-Stained Standard (Invitrogen, Carlsbad, CA, USA). After transfer, membrane was blocked with 5% non-fat dry milk at room temperature.  Membrane was incubated with swine polyclonal anti-novel H1N1 HA for two hours, washed three times, followed by incubation with goat anti-swine IgG horseradish peroxidase conjugate (ImmunoJackson Research Laboratories, Inc, West Grove, PA, USA) for one hour, and washed three times.  Detection was performed using TMB substrate (KPL, Gaithersburg, MD, USA).

### Direct Antigen Capture ELISA

Unknown samples, negative controls,  and purified novel H1 protein (Protein Sciences, Meriden, CT, USA) were directly captured to NUNC Maxisorp (Rochester, NY, USA) 96-well microplates by diluting with capture buffer (50 mM Carbonate/Bicarbonate, pH 9.6) and incubated overnight at 4°C (100 µl/well).  The microplates were washed four times with wash buffer (20 mM Phosphate Buffered Saline, 0.05% Tween-20, pH 7.2). The plates were blocked with 1.25% non-fat dry milk in capture buffer for 1 hour at 37°C (200 µl/well).  After four washes, pig polyclonal anti-novel H1N1 HA was added to wells (100 µl) and incubated for 1 hour at 37°C (diluted 1/500 in wash buffer containing 1.25% NFDM).  Following four washes, goat ant-pig IgG-HRP labeled (Jackson ImmunoResearch, West Grove, PA, USA) was added to the wells (100 µl) and incubated for 1 hour at 37°C (diluted 1/2000 in was buffer containing 1.25% NFDM). Four final washes were performed prior to the addition of 100 µl of TMB substrate (KPL, Gaithersburg, MD, USA) and incubation at 37°C for 20 minutes.  Absorbance values were measured at 620 nm and a standard curve was plotted with the purified novel H1 protein.  Linear regression analysis of the standard curve was used to calculate the novel H1 concentrations in the unknowns.

### Animal Studies

Pigs free of swine influenza virus (SIV) and porcine reproductive and respiratory syndrome virus (PRRSV) were obtained at three weeks of age.  Pigs were randomized and separated into 4 groups of 5 pigs each (table 1).  Prior to vaccination, serum was collected and tested by the homologous hemagglutination inhibition (HI) assay against the novel H1N1 A/California/04/2009 strain to confirm negative antibody status.  Sera were collected throughout the study and tested by this same HI assay to monitor seroconversion post-vaccination.  A prime/boost vaccination schedule was followed.  The first dose of vaccine was given to pigs at approximately 4 weeks of age on day 0.  On day 21 pigs received booster vaccination, with challenge on day 47 and necropsy on day 52.  Pigs received either phosphate buffered saline (PBS) (Placebo, Group 1) or different concentrations of novel H1 HA recombinant protein (Groups 2-4, Table 1).  Pigs were challenged intratracheally with virulent A/California/04/2009 (CDC# 2009712047) at a dose of 2x10^5^ TCID_50_/ml.  Nasal swabs were collected daily for live virus isolation beginning on day of challenge and continuing until study completion 5 days post-challenge.  Pigs were weighed immediately before challenge and again at necropsy for determination of average daily gain (ADG).  At necropsy, gross lung lesion consolidation was determined by a board-certified pathologist.  Lung tissue was fixed in formalin for SIV immunohistochemistry (IHC) and histopathological analysis.  Bronchoalveolar lavage fluid (BALF) was collected from lungs for live virus isolation.  This animal study was approved by the Iowa State University Institutional Animal Care and Use Committee.

### Hemaglutination Inhibition Assay

Antibodies to influenza virus were measured by HI assay run by the University of Minnesota Veterinary Diagnostic Laboratory following standard laboratory protocol. Briefly, sera were treated with receptor-destroying enzyme, heat inactivated, adsorbed with 20% turkey erythrocytes, and centrifuged. Supernatants were then serially diluted in V-shaped well microtiter plates with an equal volume containing 4-8 agglutinating units of A/California/04/2009 and plates were incubated at room temperature before addition of 0.5% turkey erythrocytes.  Titer was defined as the reciprocal of the maximal dilution at which hemagglutination was inhibited.

### Gross Lung Lesion Scoring, Histopathology, and SIV Immunohistochemistry

A single board-certified veterinary pathologist who was blinded to group treatments, performed gross lung scoring, histopathological analysis, and SIV Immunohistochemistry (IHC) analysis.  At necropsy, each lung lobe affected by pneumonia was visually estimated, and a total percentage for the entire lung was calculated based on weighted proportions of each lobe to the total lung volume[14].  Tissue samples from the trachea and all lung lobes were collected and fixed in 10% formalin.  Tissues were routinely processed and stained with hematoxylin and eosin.  Lung samples were scored according to the method used by Vincent et al[15]. Swine influenza virus IHC was done according to the method described by Vincent et al[16].  All tissue preparation and staining was done by the Iowa State University Veterinary Diagnostic Laboratory.

### Live Virus Isolation

Live virus titers were determined from nasal swabs and live virus isolation performed on BALF samples.  Briefly, nasal swabs and BALF samples were thawed and centrifuged to remove cellular debris.  The resulting supernatant was diluted 10-fold in 96 well plates in Dulbecco’s Modified Eagle Medium (DMEM) (Gibco, Carlsbad, CA, USA) containing 1% antibiotic-antimycotic (Gibco, Carlsbad, CA, USA) and 1% L-glutamine (Mediatech, Manassas, VA, USA).  After dilutions were made, 100µl was transferred from each well into respective wells of a 96 well plate which contained a monolayer of swine testicle (ST) cells.  Plates were incubated at 37°C until no further CPE was observed, typically 3-5 days.  Wells displaying CPE were considered positive, and titers were calculated using the TCID_50_/ml method of Reed-Meunch[17].

### Statistical Analysis

Single factor analysis of variance (ANOVA) was used to analyze homologous HI titers, macroscopic and histopathological lung scores, IHC and BALF results, log10 transformed nasal swab viral titers, and ADG (JMP 8.0.1, SAS Institute Inc., Cary, NC, USA).  Statistical significance was set at p < 0.05.

## Results

### Vaccine Preparation

    The novel H1N1 HA gene was inserted into the alphavirus replicon platform according to the methods listed previously.  Nucleotide sequencing after insertion confirmed the correct HA gene sequence had been maintained throughout the cloning process.  Western blotting performed on protein lysate confirmed expression of the novel HA protein at all the varying HA doses (Figure 1) used in vaccine preparation for the animal study. The HA concentration was determined by novel HA ELISA and diluted to the specified HA concentration (Table 1).  

Figure 1: Western blot confirming recombinant HA  expression.

**Figure:**
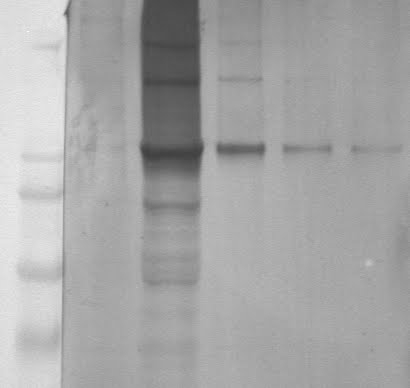

Lane 1: Ladder

Lane 2: Vero lysate (negative control)

Lane 3: Recombinant HA (28.5µg/ml)

Lane 4: Recombinant HA (1.14µg/ml)

Lane 5: Recombinant HA (0.57µg/ml)

Lane 6: Recombinant HA (0.38µg/ml)

Table 1: Design of novel H1N1 recombinant HA vaccine study.  Pigs received either placebo vaccine (PBS, Group 1) or varying doses of HA antigen (Groups 2-4). All vaccines were given intramuscularly as 2ml doses on days 0 and 21.

**Table:** 

Group	Vaccine	HA concentration/dose
1	Sham	NA
2	Recombinant HA	1.14µg
3	Recombinant HA	0.57µg
4	Recombinant HA	0.38µg

### Antibody Titers    

    Post-vaccination sera were tested for specific antibody response by the homologous HI assay.  Hemagglutination inhibition titers were not seen in vaccinated pigs after one dose, but were all positive (≥1:40), except for a single pig in Group 2, at 7 and 14 days post-boost vaccination (data not shown).  On the day of challenge, homologous HI titers were significantly higher in Groups 2-4 when compared to Group 1 (Table 2).  

Table 2.  Summary of hemagglutination inhibition (HI) titers, average macroscopic and microscopic lung involvement, immunohistochemistry (IHC), and average daily gain (ADG).

**Table:** 

Group	HI Titers^a^	% Pneumonia^b^	HistopathologicScore^c^	Lung IHC^d^	ADG^e^
1	<10	15.6 ± 5.4	1.8 ± 0.1	5/5	1.76 ± 0.2
2	121*	1.4 ± 0.9*	0.8 ± 0.2*	1/5*	2.56 ± 0.68
3	184*	0.2 ± 0.2*	0.6 ± 0.2*	0/5*	2.64 ± 0.22*
4	106*	1.8 ± 1.1*	0.8 ± 0.2*	1/5*	2.45 ± 0.34*

^a^Geometric mean homologous HI titers

^b^Group mean ± standard error

^c^0-3, group mean ± standard error

^d^Number of positive samples per group

^e^ADG post-challenge in pounds, group mean ± standard error

*Values are significantly different from non-vaccinates (Group 1) within a column at p < 0.05

### Pathological Evaluation

    At necropsy, lungs exhibited macroscopic dark purplish-red consolidated lesions located mainly in the cranioventral lobes.  Lungs taken from Groups 2-4 exhibited significantly lower lesion scores and consolidation than pigs in Group 1 (Table 2). There was also a significant reduction in pathological scores in all vaccinated groups compared to the non-vaccinated group (Table 2).  The lung sections taken from non-vaccinated Group 1 pigs had approximately 50% of the airways affected by bronchiolar epithelial disruption and peribronchiolar lymphocytic cuffing.  The vaccinated Groups 2-4 demonstrated only occasional affected airways with light cuffing.  Swine influenza virus IHC was also performed on lung sections. All 5 lungs taken from non-vaccinated Group 1 pigs were positive for influenza antigen, while only 2 pigs in total from the vaccinated Groups 2-4 were positive.   Additionally, SIV IHC was done on trachea samples taken from each pig at necropsy (data not shown).  Although there were positive trachea IHC samples in all groups, there was no significant differences between vaccinated and non-vaccinated groups.  Positive trachea IHC results correlate with what was previously reported on pathogenesis of novel H1N1 in ferrets[18].   

### Average Daily Gain

    All pigs were weighed on the day of challenge and again at necropsy.  Groups 3 and 4 had significantly higher ADG over the 5 day period following challenge than did Group 1 (Table 2).  Group 2 did exhibit higher ADG but was not significantly higher than Group 1 (p=0.08).  

### Virus Isolation

    No live influenza virus was detected one day post-challenge from nasal swabs (Table 3).  On day 2 post challenge live influenza virus was detected in Groups 1, 3, and 4, although there were no significant differences between mean group viral titers.  On day 3 post-challenge Groups 2 and 4 had significantly lower titers than did Group 1.  On both days 4 and 5 Groups 2-4 all exhibited lower titers than Group 1.  No live virus was detected in nasal swabs from any pigs in Group 2 for the duration of the challenge period.  Similarly, there was a significant reduction in the number of positive BALF samples between groups (Table 3).  By 5 days post- challenge, only a total of 3 vaccinated pigs had detectable live virus in BALF samples, while all 5 pigs in the non-vaccinated group were virus isolation positive.

Table 3. Summary of live virus isolation from nasal swabs and bronchoalveolar lavage fluid(BALF).

**Table:** 

	Nasal Swab^a^					BAL^b^
Group	1 DPC^c^	2 DPC	3 DPC	4 DPC	5 DPC	5 DPC
1	0	0.85 ± 0.53	2.55 ± 0.66	3.05 ± 0.18	3.05 ± 0.24	5/5
2	0	0	0*	0*	0*	2/5*
3	0	1.05 ± 0.07	0.65 ± 0.65	0.9 ± 0.57*	1.0 ± 0.62*	0/5*
4	0	0.45 ± 0.45	0.5 ± 0.5*	0.65 ± 0.65*	0.65 ± 0.65*	1/5*

^a^Log_10_ mean virus titers ± standard error in nasal swabs post-challenge

^b^Number of positive BALF samples per group

^c^Days post-challenge (DPC)

* Values are significantly different from non-vaccinates (Group 1) within a column at p < 0.05

## Discussion

    The recent outbreak of novel H1N1 in the human population has highlighted the zoonotic potential that influenza viruses possess.  Even before the current pandemic, there were many reported cases of swine to human transmission of influenza.  As such, part of controlling this zoonotic threat is vaccination of swine against swine influenza viruses.  In this study, we demonstrate how rapidly an efficacious swine influenza vaccine based on the alphavirus replicon expression system can be produced in response to an outbreak of a novel zoonotic strain.

      This study deomnstrated the quickness and flexibility with which a vaccine can be produced using the alphavirus replicon expression system.  It took less than two months from the time the novel HA sequence was retrieved from GISAID database until pigs were administered the first vaccine dose.  Traditional methods for producing influenza vaccines take much longer and are dependent on viral replication in embryonated eggs or on tissue culture cells with subsequent inactivation.  In the face of an influenza epidemic, a quick turnaround is important in preventing further transmission and decreasing the zoonotic potential.  The alphavirus replicon platform allows for rapid insertion of any influenza HA (or other) gene, making it an attractive influenza vaccine technology due to constant antigenic shift and drift among influenza viruses.                    

    This is the first report of immunization of swine with a recombinant protein produced via an alphavirus replicon expression system.  Replicon particle (RP) vaccines produced with this system have recently been utilized to induce protection against swine influenza virus (SIV) and porcine reproductive and respiratory syndrome virus (PRRSV) in swine[11] [19] [20].  The first proof of concept study demonstrated that a replicon particle vaccine administered to swine was able to induce high antibody HI titers against a human influenza strain. A subsequent study using an RP vaccine expressing the HA gene of a clade IV H3N2 SIV isolate confirmed that influenza HA RP vaccines given to swine are not only able to induce an antibody response, but also provide significant protection against a homologous viral challenge.  In contrast to these earlier studies, this study used an alphavirus replicon expression system to produce recombinant HA protein* in vitro*; however, similar antibody response and protection from viral challenge was demonstrated. 

    The results demonstrate that influenza infection in swine with A/California/04/2009 is able to induce clinical symptoms and gross lesions comparable to other strains of SIV[15] [21] [22]. In contrast with a previous study, several pigs (primarily in the non-vaccinated group) in this study exhibited clinical signs, mainly coughing and sneezing.  This discrepancy may be due to the miniature pig model used in the previous study[23].  In this study, vaccine administration induced specific antibody titers, reduced macroscopic and histopathologic lung lesions, and reduced viral load in both the nose and lung.  Vaccinated pigs also demonstrated a higher average daily gain than non-vaccinates. These results demonstrate that this recombinant novel HA protein is efficacious when used as a vaccine against novel H1N1 swine influenza.

     To date, novel H1N1 has been confirmed to exist in swine in several countries, including the United States[6] [7] [8].   Several recent studies have already reported the successful transmission of novel H1N1 virus from infected to naïve contact  pigs[24] [25].  The successful transmission of this virus among pigs and recent confirmation of its' presence in the United States demonstrates the need for an efficacious novel H1N1 vaccine.  This paper shows that vaccination of pigs against novel H1N1 can reduce both clinical symptoms and virus shedding in pigs, which may lead to decreased transmission.

## Acknowledgements

The authors wish to thank Drs. Matthew Erdman and J. Dustin Loy for critical reading of this manuscript.  Thanks also to Brandon Russell for his assistance, and to the Iowa State University and University of Minnesota Veterinary Diagnostic Labs, including Al Ducommun and Ling Tong.

## Funding Information

 This work was supported by the Grow Iowa Values Fund.

## Competing Interests

Harris is the founder and president of Harrisvaccines, Inc.  Vander Veen, Mogler, and McVicker are employees of Harrisvaccines, Inc.  Kamrud, Berglund, Owens, Timberlake, Lewis, and Smith are employees of AlphaVax.  Alphavax provided the novel H1N1 HA replicon vector and Harrisvaccines, Inc. provided the vaccine used in this study.  Neither Harrisvaccines, Inc. nor AlphaVax have patents associated with this replicon.  Harrisvaccines, Inc. is submitting documents to the USDA in support of conditional licensure for the vaccine. 
